# Optical and Mechanical Properties of the Multi‐Transition Zones of a Translucent Zirconia

**DOI:** 10.1111/jerd.13319

**Published:** 2024-09-26

**Authors:** Sonaj Vardhaman, Marcia Borba, Marina R. Kaizer, Do Kyung Kim, Yu Zhang

**Affiliations:** ^1^ Department of Preventive and Restorative Sciences, School of Dental Medicine University of Pennsylvania Philadelphia Pennsylvania USA; ^2^ College of Dental Medicine Columbia University New York New York USA; ^3^ University of Manchester Manchester UK; ^4^ University of Passo Fundo Passo Fundo Brazil; ^5^ Centre for Rural Dentistry and Oral Health Charles Sturt University Orange New South Wales Australia; ^6^ Post‐Graduate Program in Dentistry Universidade Positivo Curitiba Paraná Brazil; ^7^ Department of Materials Science and Engineering KAIST Daejeon South Korea

**Keywords:** ceramics, composition, fracture resistance, interface, microstructure, optical properties, prostheses, zirconia

## Abstract

**Objective:**

To characterize the composition, flexure resistance, and optical properties of a multilayer translucent zirconia in relation to its multi‐transition zones.

**Materials and Methods:**

A multilayer zirconia (5Y/4Y) and a conventional 3 mol% yttria partially stabilized zirconia (3Y) were investigated. Bar‐shaped specimens were obtained from the enamel and dentin layers, and the vertical cross‐section of 5Y/4Y (*N* = 10). A four‐point flexural (*σ*_*f*_) test was performed using a universal testing machine (1.0 mm/min). Plate‐shaped specimens (*N* = 6) were also produced from the enamel, transition 1, transition 2, and dentin layers. Translucency parameters (TP_ab_ and TP_00_) were determined using a dental spectrophotometer (*N* = 6). X‐ray fluorescence and X‐ray diffraction techniques were used to analyze elemental (*N* = 2) and phase compositions (*N* = 2), respectively. Data were analyzed using analysis of variance (ANOVA) and Tukey's test (*α* = 0.05).

**Results:**

The yttrium content and *σ*_*f*_ varied between layers of 5Y/4Y. 3Y had the highest *σ*_*f*_, followed by dentin. Enamel and cross‐section showed lower and statically similar *σ*_*f*_. 3Y and dentin groups had similar but statistically lower TP_ab_ and TP_00_ than the enamel.

**Conclusions:**

Different layers of multilayered zirconia have distinct compositions, which affect their mechanical and optical properties. The weak enamel layer compromises the mechanical properties of cross‐sectional specimens.

**Clinical Significance:**

The development of novel cubic‐containing multilayer zirconia ceramics to produce monolithic restorations brings new challenges to dental clinicians and laboratory technicians. The CAD/CAM design of multilayered 5Y/4Y restorations should consider the esthetic and mechanical requirements of each clinical case, as different properties are found in the different layers of these materials.

## 1 Introduction

Monolithic restorations have become widely used in dentistry, as they enable the complete digital workflow for time‐efficient and cost‐effective treatment [1, 2]. Zirconia is an attractive material for producing monolithic restorations due to its excellent mechanical properties and ever improving optical properties [2, 3, 4, 5, 6, 7]. Zirconia has three principal crystal structures: monoclinic at room temperature, tetragonal above 1170^o^C, and cubic above 2370^o^C. To achieve enhanced strength and toughness, the tetragonal phase is stabilized in room temperature by incorporating dopants, such as yttria (Y_2_O_3_). As a result, high toughness 3 mol% yttria‐stabilized tetragonal zirconia polycrystals (3Y‐TZP) have been developed for dental applications. 3Y‐TZP benefits from a phase transformation toughening mechanism, in which tetragonal‐to‐monoclinic phase transformation is induced by external stresses, leading to expansion of the individual grains, thereby absorbing energy and resisting crack propagation [3, 8, 9, 10].

In order to increase the translucency of this first generation dental 3Y‐TZP, modifications on the composition and microstructure were made [11]. Improved translucency was first achieved by reducing the content of alumina sintering additives and increasing the material bulk density, leading to the development of second generation 3Y‐PSZ (partially stabilized zirconia, owing to its significantly higher amount of cubic contents relative to the first generation 3Y‐TZP material) [11, 12, 13]. Although the high mechanical properties of first generation 3Y‐TZP were preserved, the second generation zirconia had only a moderate increase in translucency [3, 14, 15, 16]. Thus, its clinical indication is predominantly for monolithic crowns and bridges located in the posterior areas of the mouth and long span fixed dental prostheses (FDPs) where demands for mechanical properties are high and esthetics are relatively low.

The third generation of zirconia was produced by adding greater amounts of yttria content to increase the fraction of the cubic phase, resulting in 4, 5, and even 6 mol% yttria partially stabilized zirconias: 4Y‐PSZ, 5Y‐PSZ, and 6Y‐PSZ [17]. Increasing the optically isotropic cubic phase progressively increased the translucency [16, 18, 19], but reduced the mechanical properties relative to the first and second generations of zirconia [13, 15, 18, 20]. Studies have showed that cubic‐containing zirconias have lower flexural strength, fracture toughness, fatigue resistance, greater susceptibility to occlusal‐like sliding contact fatigue [13, 15, 18, 21, 22, 23], and a more severe wear pattern [13, 24] than previous generations of 3Y zirconias, while possessing similar elastic modulus and hardness [3] but greater translucency [3, 16].

To produce a restoration with a more natural‐looking appearance, the fourth and fifth generations of multichromatic zirconia with a layered structure have been developed [24, 25, 26, 27]. The fourth generation has contiguous layers with various pigments and/or yttria contents, which result in different shades, translucencies, and flexure resistance for different layers [17, 25]. Such microstructural and compositional variations of the material have made it possible to designate a stronger and tougher material in the gingival area and a more translucent one in the incisal edge of the restoration. However, due to their layer stacking production method, the interface between different layers often contains larger impurities and poorly sintered regions that ultimately compromise interfacial fracture resistance [26]. To address the poor fracture resistance of an abrupt interface between the layers, the fifth generation multilayer zirconia with composition and strength gradients at the interfaces has been developed [28, 29, 30, 31].

The development of these novel cubic‐containing multilayer zirconias with multi‐composition and multi‐microstructure brings new challenges for the researchers, laboratory technicians, and clinicians. A previous study concluded that the presence of different layers had no effect on the wear behavior of fourth generation zirconia [24]. On the other hand, literature reported different mechanical properties for the different layers [32, 33]. In addition, an investigation concluded that the CAD/CAM cutting depth of a multilayer zirconia puck affects the translucency and post‐fatigue load‐bearing capacity of restorations [34]. Therefore, restorations can be milled at various CAD/CAM cutting depths depending on the clinical requirements.

Clinical studies on monolithic single crowns produced with first and second generations of predominantly tetragonal‐containing zirconia reported high success rates [1, 2, 35]. Nevertheless, only a few clinical reports are available on cubic‐containing translucent zirconias [36, 37]. Therefore, a comprehensive in vitro characterization of these novel multilayer zirconias could support its indication and predict the clinical performance. Thus, the objective of this study is to investigate the chemical composition (yttria content and crystal phase composition), flexure resistance (flexural strength), and translucency parameter (TP_ab_, TP_00_, and CR) of a translucent fourth generation multilayer zirconia dental ceramic in relation to its multi‐transition zones. The study hypothesis is that different layers of the multilayer zirconia show different compositions, translucencies, and flexure resistances.

## 2 Material and Methods

The elemental composition, phase assembly, flexural strength, and translucency of a multilayer translucent zirconia ceramic (5Y/4Y, IPS e.max ZirCAD Multi, Ivoclar Vivadent) and a conventional 3Y‐PSZ (3Y, IPS e.max ZirCAD LT, Ivoclar Vivadent) control were characterized.

### 2.1 Specimen Preparation

**FIGURE 1:**
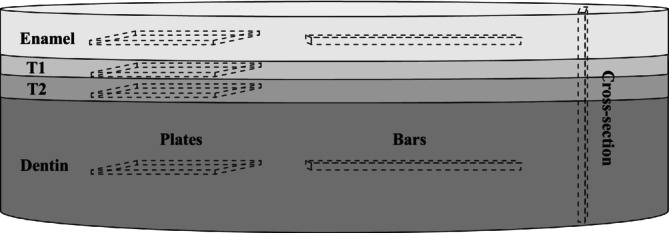
Diagram of a commercial 5Y/4Y CAD/CAM puck (IPS e.max ZirCAD Multi, Ivoclar Vivadent) depicting the sectioning patterns used to produce plate‐shaped specimens for translucency (*N* = 6), *CR* (*N* = 6), and elemental (*N* = 2) and phase (*N* = 2) composition measurements of the enamel, T1, T2, and dentin layers, as well as bar‐shaped enamel (*N* = 10), dentin (*N* = 10), and cross‐section (*N* = 10) specimens for the flexural strength test.

Three commercial CAD/CAM pucks (∅98.5 × 21 mm) of each 5Y/4Y and 3Y materials were obtained. To determine the elemental composition, phase assembly, and translucency of these materials, the 5Y/4Y pucks were cut into plate‐shaped specimens (15 × 15 × 2.5 mm) using a low‐speed diamond saw (Isomet low‐speed saw, Buehler, USA). Six plates were obtained from each layer. For enamel and dentin layers, specimens were cut from the top and bottom of the puck. For transition layers, the middle part of the puck was used; transition zone 1 (T1) was the surface near the enamel layer, and transition zone 2 (T2) was the surface near the dentin layer (Figure 1). Six plate‐shaped specimens (~15 × 15 × 2.5 mm) from the 3Y control group were also prepared using the low‐speed diamond saw. All specimens were ground using SiC abrasive papers with 320‐grit and then 800‐grit to obtain flat surfaces while removing surface damages induced from diamond saw cutting.

To measure the flexure resistance of these materials, bar‐shaped specimens were obtained. Ten bar‐shaped specimens of each group (*N* = 10) were prepared using a low‐speed diamond saw to the dimensions of 21 × 3 × 2.5 mm to compensate for the ~20% sintering shrinkage. For 5Y/4Y, the specimens corresponding to the different layers of the material were obtained by cutting the top (enamel), bottom (dentin), and the cross‐section (transition layer) of the zirconia puck (Figure 1). For the 3Y control, bar‐shaped specimens (21 × 3 × 2.5 mm, *N* = 10) were cut in the directions parallel and perpendicular to the axis of the puck. Specimens were ground using SiC papers with 320‐grit and then 800‐grit.

All specimens, both plates and bars from 5Y/4Y and 3Y materials, were sintered at 1500°C for 2 h. The top and bottom surfaces of the plates and one 2‐mm wide surface of the bars were polished down to a 1‐μm diamond suspension finish (Ecomet Polisher, Buehler, USA). The four edges of the bar specimen were chamfered [38]. The final dimension for the plate‐shaped specimens was 12 × 12 × 1 mm, and for the bar‐shaped specimens, it was 16.6 × 2.0 × 1.5 mm.

### 2.2 Elemental and Phase Compositions

For the determination of elemental composition, sintered plate‐shaped specimens (*N* = 2) from each of the four layers of 5Y/4Y as well as the 3Y control were analyzed using X‐ray fluorescence (XRF, ZSX Primus II, Rigaku, Japan). Scanning was performed using X‐ray of 60 kV voltage and 50 mA current. Chemical elements were traced from B (atomic number 5) to U (atomic number 92).

For the determination of phase contents, another set of sintered plate‐shaped specimens (*N* = 2) from 5Y/4Y and 3Y were analyzed using an X‐ray diffractometer with CuKα radiation (X'Pert3 Powder, PANalytical, Netherlands). The scanning step size was set at 0.01° with a 0.3 s dwell time over a diffraction angle range 2*ϴ* = 20° to 80°. The phase fractions of each material were quantified with Rietveld refinement full‐profile fitting using HighScore Plus [39].

### 2.3 Flexural Strength

**FIGURE 2:**
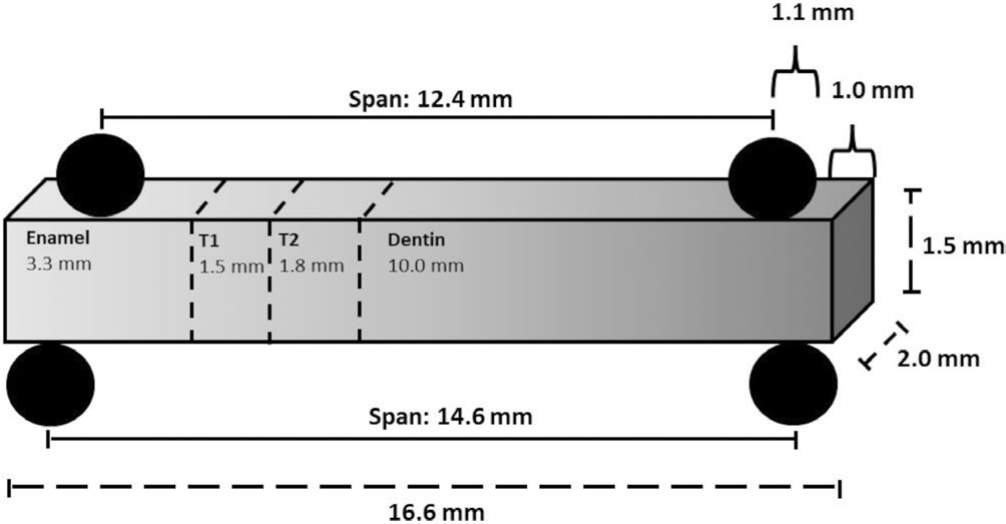
Schematic representation of four‐point flexural test.

Flexural strength was measured using the four‐point bending test with equations derived from the beam theory and validated by finite element analysis (FEA) [40]. The width (~2.0 mm) and thickness (~1.5 mm) of the specimens were measured from the center of each specimen using a digital caliper. The test was performed following the configuration described in Figure 2, aiming to introduce uniform tensile stresses across all layers and interfaces of the cross‐section specimens [26]. In order to probe the interfaces, the specimens were produced with a smaller dimension than that recommended by ISO 6872 [41]. The testing setup was previously validated using experimental and FEA predictions [26].

Specimens were tested using a universal testing machine (Model 5566, Instron Corp., Norwood, MA) with a load cell of 10,000 N and a crosshead speed of 1 mm/min. The load at fracture was recorded (*F* in N), and the flexural strength (*σ*_*f*_ in MPa) was calculated using Equation (1).

(1) $$\sigma_{f}=\frac{1.5\;F\left(l-a\right)}{bd^{2}}$$

where *b* is the specimen width (mm), *d* is the specimen thickness (mm), *a* is the distance between upper rollers (12.4 mm), and *l* is the distance between the lower rollers (14.6 mm).

After the mechanical test, all fracture surfaces were analyzed using an optical microscope (Leica MZAPO Stereo microscope, Leica Microsystems) to identify the fracture origin based on fractographic features. The fracture surface of a few specimens was also analyzed using an environmental scanning electron microscope (EVO 50, Carl Zeiss AG, Oberkochen, Germany) in low vacuum, which does not require a conductive coating.

Flexural strength data passed normality (*p* = 0.115) and equal variance tests (*p* = 0.154). Data were analyzed using one‐way analysis of variance (ANOVA) and Tukey's test (*α* = 0.05).

### 2.4 Translucency

Six sintered plate‐shaped specimens from each group (*N* = 6) were used to measure translucency parameters and contrast ratio (CR) with a calibrated dental spectrophotometer (SpectroShade TM Micro). A drop of refractometer liquid (*n* = 1.8) (Gem Refractometer Liquid, Cargille Laboratories, Inc., NJ) was placed between the specimen and background to minimize optical discontinuities. Measurements were carried out on standard white (L* = 95.7, a* = −1.3, b* = 2.6) and black (L* = 1.8, a* = 1.3, b* = −1.5) backgrounds.

Color coordinates were measured according to the CIE L*, a*, b* color space, defined by the Commission Internationale de l'Eclairge. In this color space system, L* is the lightness coordinate, while a* and b* are coordinates in the red‐green and yellow‐blue axis, respectively [42]. The translucency parameter (TP_ab_) was calculated using Equation (2), where the subscript “*W*” denotes the coordinates read on a white background and “*B*” on a black background.

(2) $${\text{TP}}_{\text{ab}}=\sqrt{{\left({L^{*}}_{B}-{L^{*}}_{W}\right)}^{2}+{\left({a^{*}}_{B}-{a^{*}}_{W}\right)}^{2}+{\left({b^{*}}_{B}-{b^{*}}_{W}\right)}^{2}}$$

The translucency parameter (TP_00_) was also calculated using the CIE*ΔE*2000 (1:1:1) color difference formula [43], according to Equation (3).

(3) $$\begin{array}{cc} {\mathrm{TP}}_{00}= & \left[{\left(\frac{L\prime_{B}-L\prime_{W}}{K_{L}\;S_{L}}\right)}^{2}+{\left(\frac{C\prime_{B}-C\prime_{W}}{K_{C}\;S_{C}}\right)}^{2}+{\left(\frac{H\prime_{B}-H\prime_{W}}{K_{H}\;S_{H}}\right)}^{2}\right.\\ & {\left.+,R_{T},\left(\frac{C\prime_{B}-C\prime_{W}}{K_{C}\;S_{C}}\right),\left(\frac{H\prime_{B}-H\prime_{W}}{K_{H}\;S_{H}}\right)\right]}^{1/2} \end{array}$$

where the subscripts “*B*” and “*W*” refer to lightness (*L*′), chroma (*C*′), and hue (*H*′) of the specimens over the black and the white backgrounds, respectively. Weighting functions, *S*_*L*_, *S*_*C*_, and *S*_*H*_, adjust the total color difference for variation in the location of the color difference pair in a specimen over the *B* and *W* backgrounds in *L'*, *a'*, *b'* coordinates. *K*_*L*_, *K*_*C*_, and *K*_*H*_ are correction terms for experimental conditions. *R*_*T*_ is the rotation function that accounts for the interaction between chroma and hue differences in the blue region.

The CR was defined as the ratio of the spectral reflectance of the light (*Y*) of the test specimen when it is placed over a black background (*Y*_*B*_) to a white background (*Yw*), according to Equations (4) and (5). CR of 0 represents a transparent material, while CR of 1 represents a totally opaque material. The specified white stimulus *Y*_*n*_ is equal to 1.00 [44].

(4) $$\mathrm{CR}=\frac{Y_{B}}{Y_{w}}$$

(5) $$Y={\left(\frac{L^{*}+16}{116}\right)}^{3}Y_{n}$$

TP_ab_, TP_00_, and CR data passed normality and equal variance tests and were analyzed using one‐way ANOVA and Tukey's post hoc test (*α* = 0.05).

## 3 Results

### 3.1 Elemental and Phase Compositions

**FIGURE 3:**
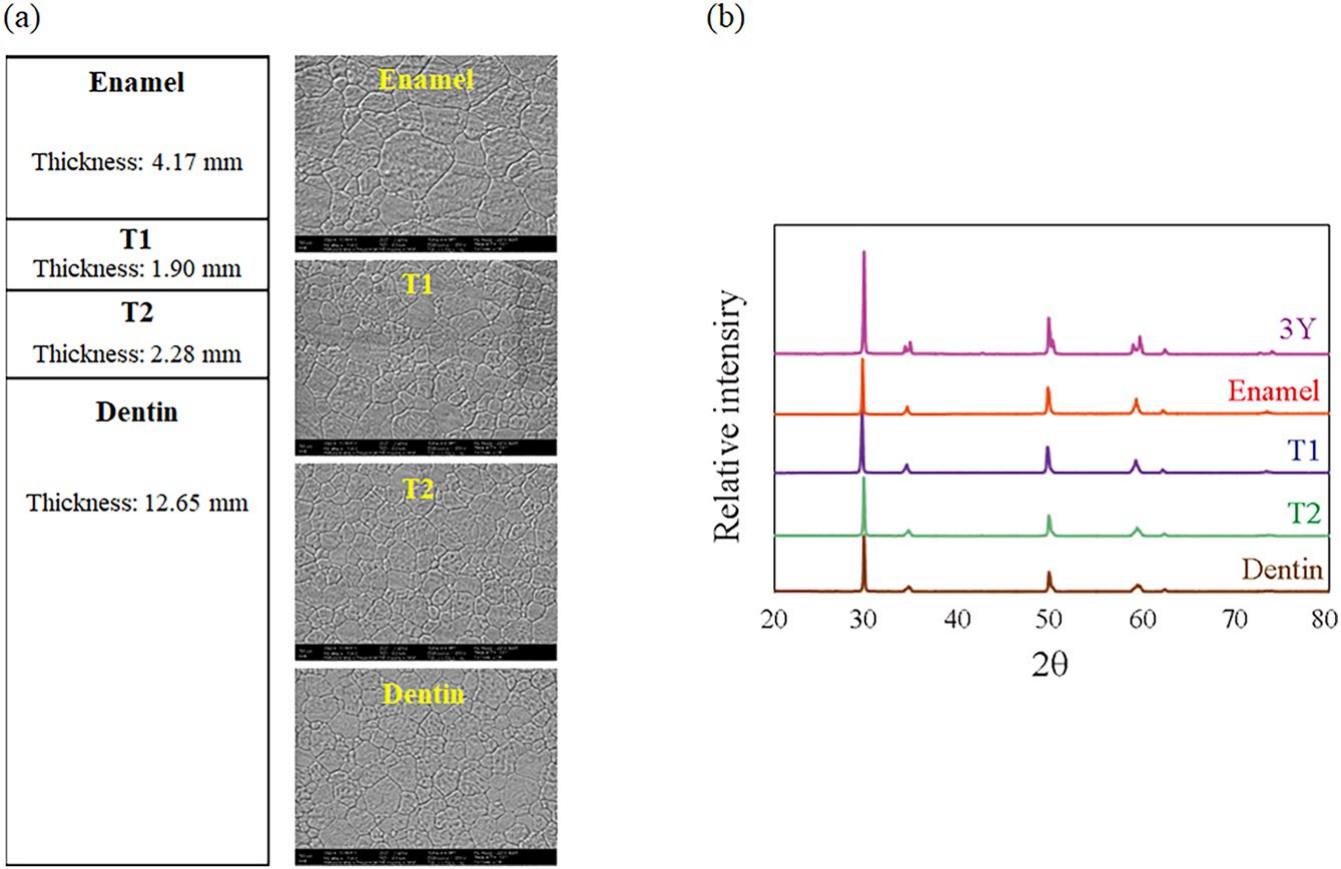
Microstructure and crystallographic structure. (a) Schematics of a 5Y/4Y multilayer zirconia: The respective thicknesses of different layers in a ∅98.5 × 21 mm CAD/CAM puck and representative FE‐SEM images of each layer. (b) XRD spectra of the different layers of 5Y/4Y and 3Y control.

The yttrium content was different among the four layers of the 5Y/4Y material. The enamel layer showed the highest yttrium content of 7.1 wt% (equivalent to 5.1 mol%). The transition zones T1 and T2 had 6.7 wt% (4.8 mol%) and 6.2 wt% (4.5 mol%), respectively. The dentin layer showed the lowest yttrium content, about 5.6 wt% (4 mol%). Other trace elements, which are commonly used as pigments like Al, Fe, and Ti, were found in similar proportion in all layers. The yttrium content for 3Y was 5.3 wt% (3.0 mol%). The microstructure and compositions of the test materials are presented in Table 1 and Figure 3, respectively.

**TABLE 1:** Microstructure and compositions of the test materials.

Material	Structure	Y_2_O_3_ wt%^a^	Y_2_O_3_ mol%^b^	Cubic content wt%^c^	Mean grain size (standard deviation) ‐ μm^d^
3Y (IPS e. max ZirCAD LT)	Monolith	5.3	3.0	15	1.08 (0.23)
5Y/4Y (IPS e. max ZirCAD Multi)	Enamel	7.1	5.1	80	1.87 (0.35)
	T1	6.7	4.8	64	1.72 (0.32)
	T2	6.2	4.5	49	1.40 (0.15)
	Dentin	5.6	4.0	46	1.12 (0.21)

^a^ Measured using the X‐ray fluorescence (XRF) method.

^b^ Calculated using the XRF data and by assuming that all compositions contain 0.05 wt% Al_2_O_3_.

^c^ Measured using the XRD method.

^d^ Measured using the linear intercept method.

X‐ray diffraction (XRD) spectra from the four layers of 5Y/4Y and 3Y materials are shown in Figure 3b. Rietveld refinement analysis revealed only tetragonal and cubic phases in these materials. The cubic contents were 80, 64, 49, and 46 wt% in 5Y/4Y enamel, T1, T2, and dentin, respectively, and 15 wt% in 3Y control.

### 3.2 Flexural Strength

There were significant differences among the mean flexural strength values for the test groups (*p* < 0.001). 3Y control had the highest mean flexural strength, followed by the 5Y/4Y dentin group. 5Y/4Y enamel and cross‐section groups showed lower but statically similar mean flexural strength values (Table 2).

**TABLE 2:** Mean values (standard deviation) of flexural strength (*σ*_*f*_), translucency parameters (TP_ab_ and TP_00_), and contrast ratio (CR) for the experimental groups.

Groups	*σ*_*f*_	TP_ab_	TP_00_	CR
3Y	851.1 (96.8)^a^	26.27 (0.05)^b^	19.38 (0.02)^b^	0.51 (0.00)^a^
Dentin	744.2 (103.8)^b^	28.96 (0.71)^b^	20.32 (0.56)^b^	0.49 (0.01)^a^
Enamel	386.7 (59.8)^c^	36.74 (1.72)^a^	27.70 (1.28)^a^	0.35 (0.02)^b^
Cross‐section	429.8 (54.0)^c^	—	—	—
*p*	< 0.001	< 0.001	< 0.001	< 0.001

*Note:* Means followed by similar letters in the same column are statistically similar (*p* ≥ 0.05).

**FIGURE 4:**
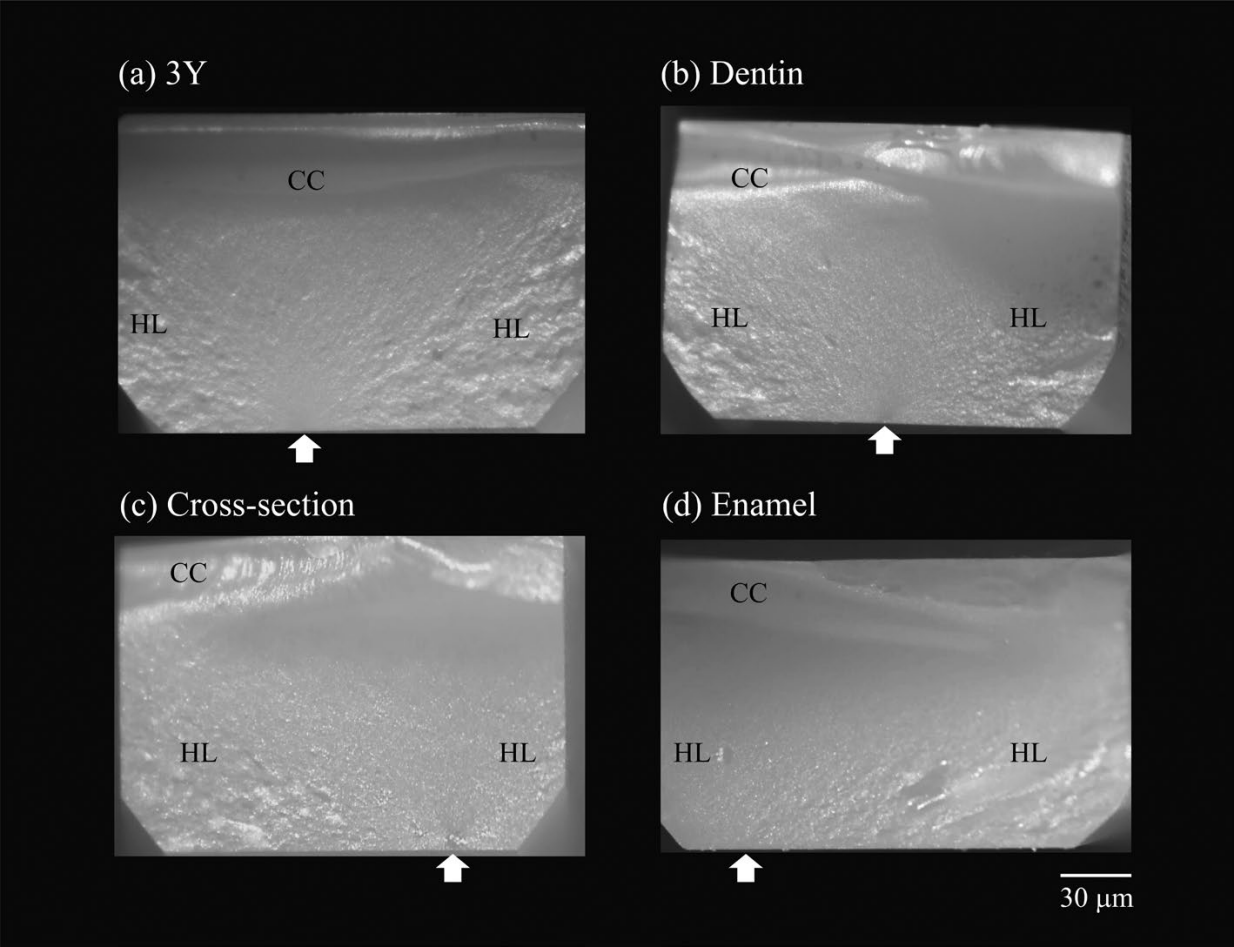
Images of the fracture surface of specimens from groups: (a) 3Y, (b) dentin, (c) cross‐section, and (d) enamel. Compression curl (CC) and hackle lines (HL) indicate the direction of crack propagation. White arrows point to the fracture origin.

**FIGURE 5:**
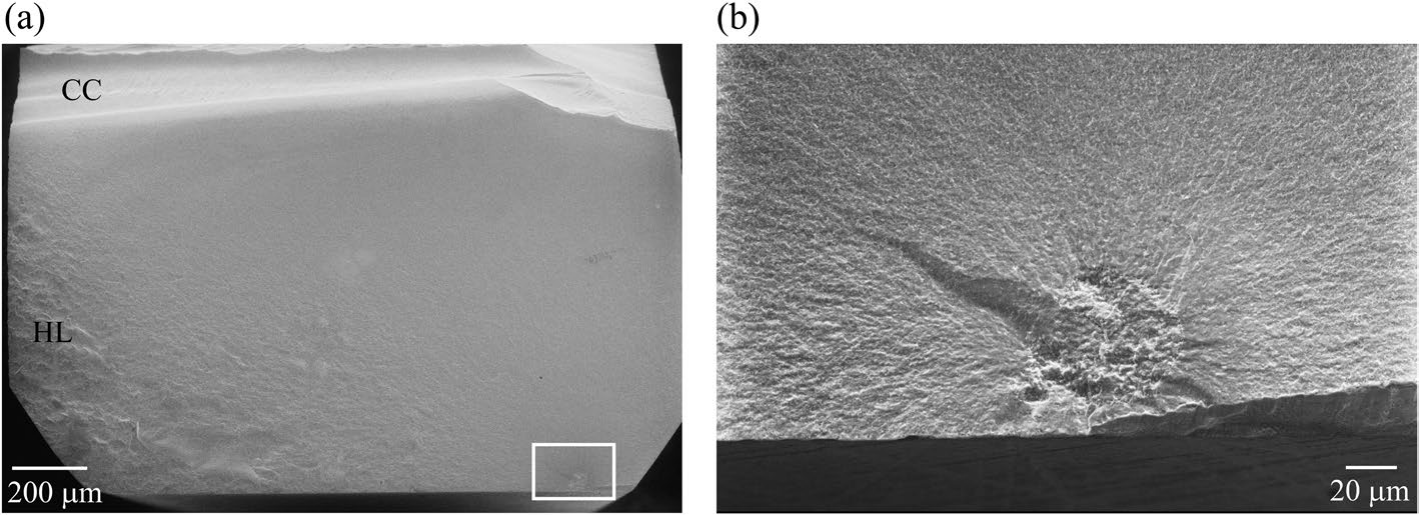
(a) SEM images of the fracture surface of a specimen from the cross‐section group (also shown in Figure 4c) that failed from the dentin layer showing a large flaw indicated by the white box (b). Compression curl (CC) and hackle lines (HL) indicate the direction of crack propagation.

Based on fractographic features such as compression curl and wake hackles, it was possible to identify the fracture origin, which was typically located at or near the ceramic tensile surface for all test groups (Figure 4). For cross‐section specimens, 50% failed at the enamel layer, 30% between T1 and T2, and 20% at dentin. A large internal defect was identified as the fracture origin for specimens that failed at the dentin layer, as shown in Figure 5.

### 3.3 Translucency

There were significant differences among groups for TP_ab_, TP_00_, and CR (*p* < 0.001) (Table 2). 3Y control and 5Y/4Y dentin layer had similar and statistically lower TP_ab_ and TP_00_ mean values than the enamel layer.

For CR, 3Y and dentin layer showed similar and significantly higher mean values than the enamel layer.

## 4 Discussion

Novel multilayered translucent zirconias were developed in an effort to produce monolithic restorations with good mechanical performance and tooth‐like esthetics [24, 27, 28, 29, 30, 31, 32, 33, 34]. In the present study, the composition, microstructure, and properties of a fourth generation multilayer zirconia have been analyzed in detail. Different compositions, flexure resistance, and translucency were found for the distinct layers of the multilayer zirconia, agreeing with the study hypothesis. The yttrium content, within the multilayer material, decreases gradually from the enamel to the dentin layer. Moreover, the 3Y control group had the lowest yttrium content. Studies have shown that an increase in yttrium content results in an increase in the cubic phase fraction [3, 11, 15, 27]. Additionally, a previous investigation reported, for the 5Y/4Y material, a higher cubic content and larger grain size of the enamel layer and its adjacent transition zone 1 (T1) relative to transition zone 2 (T2) and the dentin layer [24]. These findings suggest that the enamel layer is composed of 5Y‐PSZ and the dentin layer is composed of 4Y‐PSZ, which corroborated with the information provided by the manufacturer.

The differences in the composition of the distinct layers of 5Y/4Y affected their mechanical and optical properties. The enamel layer showed lower flexural strength than the dentin layer, mainly due to its higher yttrium content and cubic phase fraction and larger grain size. Increasing the cubic content of zirconia ceramics could hinder the tetragonal‐to‐monoclinic transformation toughening mechanism and decrease its fracture resistance and toughness [3, 13, 18, 22, 27]. The same rationale can explain the superior strength of 3Y control in comparison to dentin (4Y‐PSZ) and enamel (5Y‐PSZ) layers of 5Y/4Y.

Characterizing these multi‐composition multi‐microstructure materials represents a challenge, considering that, clinically, the entire multilayer structure (restoration) will be subjected to mechanical stresses. Dental prostheses are subjected to stresses that are more complex than the ones induced by standard flexural strength tests [45], which is a study limitation. Yet, the production process of multilayer ceramics is likely to create interfacial defects, making the interfaces more susceptible to fracture and affecting the ceramic structural performance [26]. Therefore, cross‐section specimens were produced, including the four layers of the CAD/CAM puck, and the 4‐point flexural strength test was designed to induce uniform tensile stresses in all layers and interfaces [26]. An interesting finding was the fact that these cross‐section specimens showed similar flexural strength to that of the enamel group. In addition, fractographic analysis showed that only 20% of the cross‐section specimens failed from the dentin layer, and these failures were associated with abnormally large internal defects (Figure 5). Most specimens failed from surface and near‐surface flaws located at the enamel layer and the transition zones, meaning that the weaker high‐yttrium content and high‐cubic fraction layers are more susceptible to flexural fracture of the cross‐section specimens.

A study reported greater flexural strength, fracture toughness, and fatigue performance for specimens produced from the dentin layer (4Y‐PSZ) in comparison to the enamel layer (5Y‐PSZ) of 5Y/4Y, which corroborates with the current findings, while specimens produced from the transition zones had intermediate values [33]. When monolithic and multilayer zirconias were evaluated using a more realistic crown‐shaped configuration, it was possible to demonstrate that the yttria content at the occlusal area determined their fracture resistance, regardless of the yttria content at the other areas of the crown [27]. Moreover, an investigation reported a significant effect of the CAD/CAM cutting depth of the multilayered puck on the load‐bearing capacity of the prosthetic crowns [34].

The layered manufacturing technique could also compromise the bulk strength of the material, as observed in a previous study that evaluated the mechanical behavior of another fourth generation multilayer zirconia [26]. The fourth generation zirconia multilayered pucks, including the ones used in the present study, are commonly produced by pressing powders of different compositions in increments, which could lead to flaws that may weaken the interfaces. Yet only 30% of cross‐section specimens failed at the transition zones, and the strength values obtained were in the same range of the enamel layer, suggesting the absence of large interfacial flaws, which was confirmed by the fractographic analysis.

More recently, a gradient technology, based on the blend of two zirconium oxide raw materials, was used by the manufacturer to produce a material with a “layer‐free” transition zone between dentin and enamel (i.e., the fifth generation multilayer zirconia) [27, 32]. A study compared the fracture resistance of crowns produced from the fourth and fifth generation multilayer zirconia after aging and found similar mechanical behavior [27]. Nevertheless, most studies on these novel graded zirconias either characterized the properties of the individual layers separately [29] or evaluated the fracture resistance of crowns and FDPs made from these materials [27, 28]. Further definitive studies are recommended to verify if modifications in the manufacturing process truly improved their mechanical performance.

A higher yttrium content and cubic phase fraction in the enamel layer also led to greater translucency relative to the dentin layer and 3Y control group. The optical behavior of zirconia can be progressively improved by increasing the content of optically isotropic cubic phase, which also has a larger grain size, reducing light scattering at grain boundaries [11, 16, 18]. Therefore, the CAD/CAM design of 5Y/4Y restorations should consider the esthetic and mechanical requirements of each clinical case [28, 34]. Posterior crowns and FDPs' connectors are subject to greater stresses and should be milled at lower regions of the zirconia puck, where better mechanical properties are found. On the other hand, anterior restorations can be milled at weaker but more translucent regions. Nonetheless, clinical extrapolations should consider that materials were characterized using bar and plate‐shaped specimens, meaning that the effect of the prosthesis design was neglected. Furthermore, oral environment simulations, such as fatigue testing, should be included in future investigations.

## 5 Conclusions

The different layers of multilayered zirconia have distinct compositions, which affect their mechanical and optical properties. The dentin layer has lower yttrium content, greater flexural strength, and lower translucency than the enamel layer. The weaker enamel layer compromises the mechanical properties of cross‐section specimens.

## Conflicts of Interest

The authors declare no conflicts of interest.

## Data Availability

The data that support the findings of this study are available from the corresponding author upon reasonable request.
